# Temporal changes in health-related lifestyle during the COVID-19 epidemic in Finland – a series of cross-sectional surveys

**DOI:** 10.1186/s12889-022-14574-y

**Published:** 2022-11-19

**Authors:** Tuija Jääskeläinen, Tommi Härkänen, Peppi Haario, Elina Isosaari, Annamari Lundqvist

**Affiliations:** Finnish Institute for Health and Welfare, P.O. Box 30, FI-00271 Helsinki, Finland

**Keywords:** Lifestyle, COVID-19 epidemic, Diet, Sleep, Physical activity, Smoking, Alcohol consumption

## Abstract

**Background:**

Public health recommendations and governmental restrictions during the COVID-19 epidemic have affect everyday life. This study aimed to examine temporal changes in health-related lifestyle and the accumulation of positive and negative changes in the key lifestyle factors (vegetable consumption, leisure-time physical activity, sleeping, alcohol consumption, smoking) in the same individuals among Finnish adults during the epidemic.

**Methods:**

This study was based on a series of cross-sectional surveys conducted between April 2020 and June 2021 to investigate antibody levels for the new coronavirus in the population. In each survey, a random sample of adults (18 to 69 years) from five university hospital regions were invited. A total of 5655 (response rate 32%) responded to the questionnaire including questions on lifestyle changes during epidemic.

**Results:**

On average one-sixth of respondents (17%) reported at least two negative changes in the key lifestyle factors during the study period. An increase in snacking and sleep problems and decrease in leisure-time physical activity and active commuting to work were the most common of individual negative changes. The proportion of negative changes in physical activity increased as the epidemic drags on. In contrast, on average every seventh of the respondents (14%) reported at least two positive lifestyle changes in the key lifestyle factors. The most common individual positive changes were increased consumption of fruit, berries and vegetables and decreased consumption of alcohol. More negative changes were reported on average, when both negative and positive changes in the key lifestyle factors were summed. The most negative changes were reported in the late 2020.

**Conclusion:**

The results of the present study suggest that the lifestyle changes during the COVID-19 epidemic have been diverse being on average more commonly unfavorable than favorable for health. The deteriorated epidemic situation in the late 2020 and, on the other hand, prolonged epidemic predisposed to negative lifestyle changes. Further studies are important to examine whether these changes are maintained over time and to identify the factors that contribute to changes and their accumulation in the same individuals. Health promotion actions are needed to prevent the long-term effects of the epidemic on health and welfare.

**Supplementary Information:**

The online version contains supplementary material available at 10.1186/s12889-022-14574-y.

## Introduction

The rapid spread of COVID-19 to nearly all parts of the world and measures aiming to control it have posed enormous health, economic, environmental, and social challenges worldwide [1, 2]. In the absence of effective drugs or vaccines during the first waves of the epidemic, lockdown have been introduced to reduce spread of the virus. Restrictions, like lockdowns, are an important safety measure to protect public health. Lockdown, stay at home, social distancing and travel restrictions are worldwide strategies to control spread the COVID-19, but the restrictions vary between countries and depend on the epidemic situation [3, 4]. Finland was in state of emergency due to COVID-19 from 16 March to 16 June 2020 and from 1 March to 27 April 2021 [5]. In general, restrictions during the epidemic covered for example public and private events, public premises, restaurants, and group hobbies. Further, there were recommendations and restrictions concerning educational institutions and higher education institutions as well as recommendation to work remotely [6].

The COVID-19 epidemic and its restriction measures have had comprehensive effects on health and wellbeing in the population and its subgroups. From the public health perspective, unfavorable development has been observed relating to both physical and mental health [7] as well as to quality of life [8]. Further, there is growing cross-sectional evidence from different population studies worldwide that COVID-19 epidemic has affected on key indicators of health-related lifestyle, i.e. on eating habits, physical activity, sleeping, smoking and alcohol consumption [9–19]. In general, previous studies have reported both unfavorable and favorable changes in lifestyle with variation between age-, gender and other population groups. For example, there are indications that women are more prone to lifestyle changes than men [10, 19]. Several population studies have shown increasing in unhealthy snacking [9–11, 20] but the consumption of vegetables for example, has been shown to increase and decrease (e.g.,20). Findings concerning leisure-time physical activity during the COVID-19 epidemic are mixed showing both decrease and increase in leisure-time physical activity with indications that unfavorable changes have occurred especially in vulnerable population groups [20–22]. Sleep problems, such as decreased sleep quality and sleep disturbances, have shown to increase which may have adverse effects on mental health, for example [23]. Thera are also some observations of increased smoking or alcohol use [17, 18] but, however, in some studies no changes have been observed [19].

However, despite the growing number of research publications related to lifestyle changes during the COVID-19 epidemic, more information is needed to clarify and confirm the results in different populations. Further, there are still knowledge gaps related to temporal changes in lifestyle during the epidemic. This information is needed to evaluate the impact of epidemic situation to lifestyle. In addition, to assess the public health impact related to lifestyle changes during the epidemic the accumulation of multiple negative or positive changes in the same individuals needs further investigation. Thus, we aimed to answer the following research questions: 1) what kind of changes in the key health-related lifestyle factors (i.e. diet, physical activity, sleep, smoking, alcohol consumption) are observed at different stages of epidemic in the Finnish adult population, 2) whether negative or positive changes in lifestyle during the epidemic accumulate in the same individuals, and 3) whether age- or gender specific differences are observed.

## Methods

### Study design and setting

The present study is based on the online questionnaire conducted as a part of The Serological Population Study of coronavirus epidemic [24] which examines the spread of the coronavirus using blood sampling in repeated cross-sectional surveys in Finland.

### Sampling and sample size

**Fig. 1:**
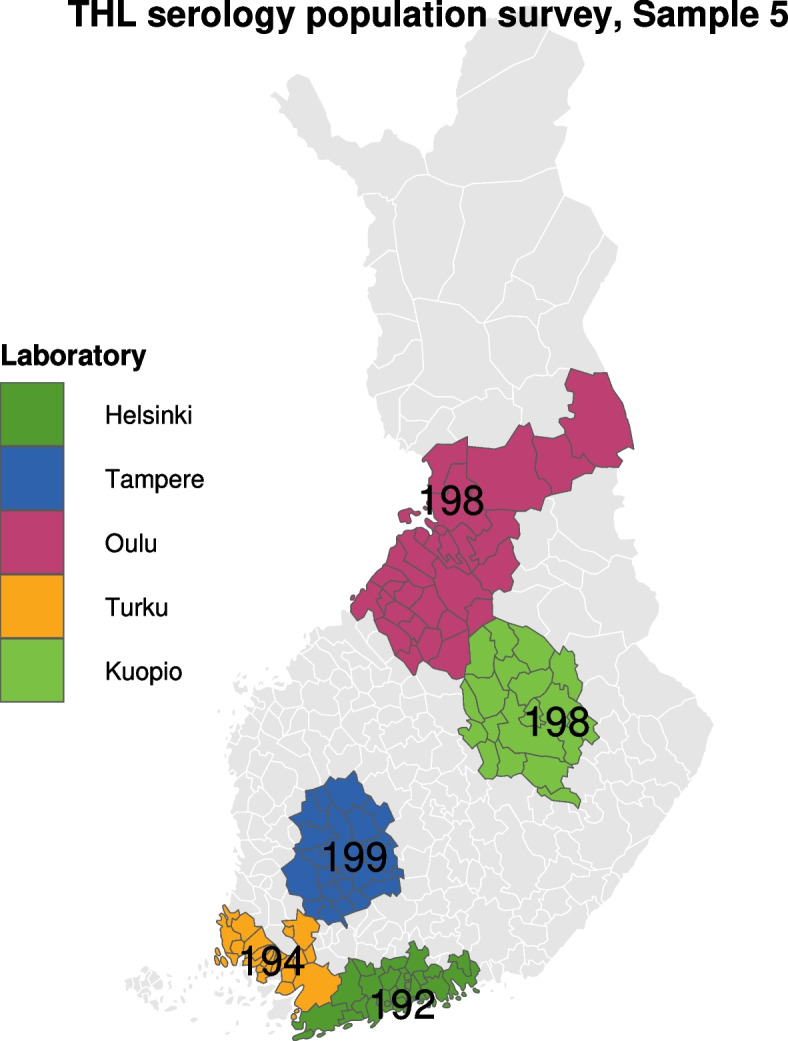
The geographical areas from which the random samples were collected. Legend: An example of the study sample 5 (*n* = 1000), sampling date 23.4.2020

The samples were selected from geographical strata (Fig. 1) defined by the surrounding areas of the five university hospitals (Helsinki, Turku, Tampere, Kuopio and Oulu) in Finland, and the calendar time of the repeated cross-sectional surveys conducted approximately every week from April 2020 to May 2020, and every 2 weeks from June 2020 to May 2021 in the Helsinki region and every 4 weeks in the other regions. The samples were drawn randomly from the strata using the Population Information System. Those living in institutions were excluded. In total 17,592 men and women aged 18–69 were invited to fill in the questionnaire between April 2020 and May 2021, and 5654 of them responded (response rate 32%).

### Data collection and questionnaire

Age, gender and geographical area were obtained from the Population Information System.

In addition to blood sampling, the participants were asked to complete an online questionnaire after the research visit. The questionnaire was planned and coordinated by the Finnish Institute for Health and Welfare (THL) which has decades of experience in conducting population-based research. The previous questionnaires conducted by THL formed the basis for the questionnaire used in the present study which was modified for the special needs to examine the impact of the COVID-19 epidemic and its restriction measures on population health and wellbeing. In the present study, eight out of nine questions on health-related lifestyle were utilized. The respondents were asked to assess whether the COVID-19 epidemic or its restrictions have had effects on alcohol consumption, smoking, leisure-time physical activity, active commuting to work, sleep problems and nightmares, unhealthy snacking (for example sweets, chocolate, soft drinks, chips) and consumption of vegetables (excluding potatoes), fruits and berries (question concerning the number of meals per day was excluded in the present study). Response alternatives were: 1) no change, 2) yes, decreased, 3) yes, increased, 4) not concern me.

In addition to examine separate changes in each lifestyle factor, the accumulation of healthy and unhealthy changes in the same individuals were examined using summary measures. For the summary measures, one indicator (i.e. vegetable consumption, leisure-time physical activity, sleep problems and nightmares, alcohol consumption, smoking) of each key lifestyle habit to reduce the risk of chronic diseases and increase overall life expectancy [25, 26], were selected. We had two different dichotomized summary measures. The first one was formed by summing up the number of negative changes in the key lifestyle factors (i.e. decreased consumption of vegetables, decreased leisure-time physical activity, increased sleep problems and nightmares, increased alcohol consumption, increased smoking) and dichotomizing it into those having at least two negative changes vs. others. The second one was formed in reverse by summing up the number of positive changes in the five key lifestyle factors and dichotomizing it into those having at least two positive changes vs. others. We excluded those with missing data on the key lifestyle factors (*n* = 136).

We also examined the changes of the five key lifestyle factors by the annual quarters. For each factor, a negative change has the value of − 1, a positive change + 1 and no change or not concern me 0. The sum of changes varied between − 5 and + 5 points. For example, if the response to all five key lifestyle factors was a negative change, the respondent had − 5 points and if the response to all factors was a positive change, the respondent had + 5 points. In these analyses, we excluded individuals with missing data on any of these factors (*n* = 136) and those with no changes in any of the five studied lifestyle factors (*n* = 87).

Education (in years, categorized for the analyses using the tertiles) and the number of family members (categorized as living alone or not) were also used. Due to the limited number of respondents, the responses received during the study period were divided into five annual quarters based on the date when the subjects responded to the questionnaire (I April–June 2020, II = July–September 2020, III=October–December, IV = January–March 2021 and V = April–June 2021).

### Data analysis

The inverse probability weights (IPW) based on registry-based information on age, gender, sampling date and geographical area were created to minimize the impact of non-response and attrition [27–29]. The weights were calibrated to the sample in each stratum based on the geographical areas and sampling date. The stratification, and the IPW were incorporated in all analyses using the R software (version 3.6.3.) [30] and its survey package [31]. As unadjusted descriptive statistics, the prevalences for decrease and increase in the lifestyle habits were estimated in each annual quarter. Further, the prevalences and their 95% confidence intervals were estimated for dichotomized summary measures (i.e at least two negative or at least two positive changes) for all respondents and stratified by gender and age group (18–49, 50–69 years). Finally, the average score of the sum of changes and its 95% confidence intervals were estimated. As age and gender adjusted analyses, we tested whether there were linear trends over the study period using generalized linear models.

### Ethical considerations

The study was approved by the Ethics Committee at the Hospital district of Helsinki and Uusimaa, and all participants provided their written informed consent prior to the commencement of the study.

## Results

The proportion of women was higher among the respondents (61%) than among nonrespondents (45%) indicating that men responded considerably less frequently (Supplementary Table 1). Half of the respondents (53%) were aged between 18 and 49 years and other half (47%) were 50–69 years. The response rate was only 20% in the youngest age group of 18–29 years whereas in the age group 50–69 it was 40%. Also the response rate was much lower after July 1, 2020 than before it. One-third of the respondents (30%) were either low or intermediate educated, while 38% of respondents were highly educated.

**Fig. 2:**
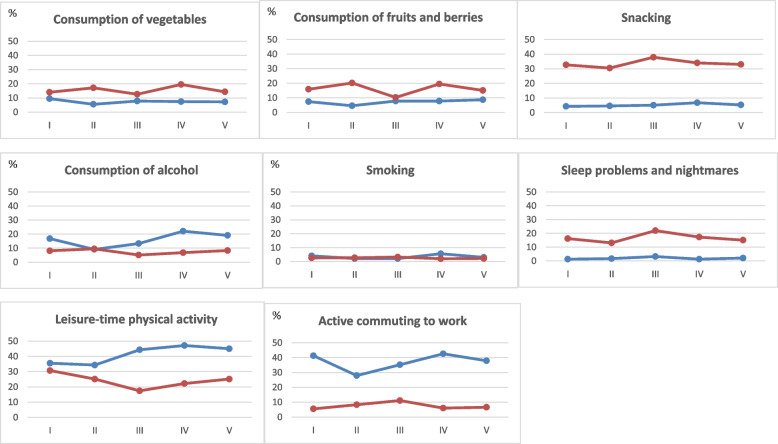
Changes in lifestyle during COVID-19 epidemic (from April 2020 to June 2021, *n* = 5655). Legend: Blue line indicates the weighted proportion of respondents who reported decrease and red line the weighted proportion who reported increase in the lifestyle habits. Time periods: I = April–June 2020, II = July–September 2020, III=October–December, IV = January–March 2021 and V = April–June 2021

On average every sixth of respondents (17%) reported at least two negative changes in the key lifestyle factors between April 2020 and June 2021 (Table 1). Only 4% of respondents reported at least three negative changes. Negative changes were occurred most often in leisure-time physical activity, which decreased remarkably (range 34–47% in the annual quarters) (Fig. 2). In addition, more than a third reported a reduction in active commuting to work (28–43%). A greater percentage of respondents also reported an increase in unhealthy snacking (31–38%) and in sleep problems and nightmares (13–22%) than a decrease (4–7%, 1–3%). Women reported slightly more often increased in snacking and sleep problems and nightmares compared to men during the study period (Supplementary Table 2).

**Table 1:** The prevalences of the dichotomized summary measure of the five key lifestyle factors by annual quartiles

The summary of lifestyle factors (***n*** = 5519)	April–June 2020	July–September 2020	October–December 2020	January–March 2021	April–June 2021	*p* for trend^**1**^
	% (95% CI)	% (95% CI)	% (95% CI)	% (95% CI)	% (95% CI)	
**≥ 2 negative changes** ***n*** **= 874**						
All	15,7 (13,9-17,5)	14,1 (10,0–18,2)	22,3 (14,9-29,7)	18,2 (15,1-21,3)	15,6 (11,7–19,5)	0,555
**Gender**						
Women	16,5 (14,3-18,7)	15,7 (10,2-21,2)	18,8 (10,8-26,8)	22,0 (17,7–26,3)	17,2 (12,3-22,1)	0,312
Men	14,9 (12,4–17,4)	12,7 (6,6–18,8)	26,0 (13,5-38,5)	14,5 (10,2-18,8)	14,3 (8,4-20,2)	0,932
**Age groups**						
18–49 years	17,6 (15,4–19,8)	15,2 (9,5-20,9)	20,5 (11,1-29,9)	19,2 (15,3-23,1)	14,9 (10,0–19,8)	0,686
50–69 years	11,6 (9,2-14,0)	12,2 (6,9-17,5)	26,0 (14,2-37,8)	16,1 (11,6-20,6)	17,6 (11,5-23,7)	0,035
**≥ 2 positive changes** ***n*** **= 756**						
All	15,2 (13,4–17,0)	12,3 (8,8-15,8)	11,7 (6,0–17,4)	17,6 (14,5-20,7)	15,0 (10,7–19,3)	0,436
**Gender**						
Women	17,5 (15,1–19,9)	13,2 (8,1–18,3)	5,9 (1,2-10,6)	16,8 (12,9-20,7)	13,7 (9,0–18,4)	0,283
Men	12,8 (10,4–15,2)	11,5 (6,4–16,6)	18,0 (7,6-28,4)	18,5 (13,2-23,8)	16,0 (9,3-22,7)	0,041
**Age groups**						
18–49 years	15,5 (13,3-17,7)	10,5 (6,0–15,0)	11,9 (4,6–19,2)	19,5 (15,2-23,8)	15,1 (9,8-20,4)	0,193
50–69 years	14,5 (12,0–17,0)	15,5 (9,4-21,6)	11,3 (2,7–19,9)	13,2 (9,1–17,3)	14,5 (8,4-20,6)	0,406
**No changes at all** *n* **= 87**						
	0,4 (0,2 − 0,6)	0,9 (−0,1-1,9)	0,7 (−0,7 − 2,1)	0,9 (0,1-1,7)	0,2 (− 0,2 − 0,6)	0,007

Weighted prevalences (%) and 95% confidence intervals (CI). The five key lifestyle factors: vegetable consumption, leisure-time physical activity, sleep problems and nightmares, alcohol consumption, smoking

^1^ Age and gender adjusted linear trend based on generalized linear models

Overall, the proportions of those reporting increase in snacking and sleep problems and nightmares remained fairly the same level from the first wave of COVID-19 epidemic (from April 2020) (Fig. 2). The proportions of those, who decreased leisure-time physical activity and active commuting to work’, increased as the epidemic drags on. In general, there were no major temporal changes in the proportion of those reporting at least two negative changes during the epidemic (Table 1). In the older age group, however, this proportion slightly increased as the epidemic drags on.

About one in seven (14%) reported at least two positive changes in the key lifestyle factors during the epidemic (Table 1). Only 3% of respondents reported at least three positive changes. Positive changes occurred most commonly in leisure-time physical activity, which increased the most (17–31%) during the epidemic. Additionally, a positive change occurred in the consumption of alcohol, which decreased (in 9–22% of the respondents) more than increased (5–10%) during the epidemic. A larger percentage of the respondents reported an increase in the consumption of fruits and berries (10–20%) and the consumption of vegetables (13–20%) than a decrease (5–9%, 6–10%) throughout the study period.

Overall, the consumption of vegetables, fruits and berries remained fairly the same level from the first wave of COVID-19 epidemic (from April 2020) (Fig. 2). Decrease in alcohol consumption became more common during the epidemic. The differences between decreasing and increasing in leisure-time physical activity were highest towards the end of the year 2020 and the beginning of the year 2021. An increasing trend was observed among men who had at least two positive lifestyle changes across the study period (*p* = 0.041) (Table 1).

The average of the sum of changes in vegetable consumption, leisure-time physical activity, sleep problems and nightmares, alcohol consumption and smoking was negative in almost all annual quarters indicating that more negative than positive changes were reported (Table 2). The most negative changes were reported in late 2020 (October–December 2020). There was a decreasing trend among women in the sum of changes over the study period (*p* = 0.022). No differences between the age or gender groups in the sum of changes could be confirmed.

**Table 2:** The average score of the summary measure of the five key lifestyle factors by annual quarters

The average scores of the summary measure (***n*** = 5432)	April–June 2020	July–September 2020	October–December 2020	January–March 2021	April–June 2021	***p*** for trend^**1**^
	**Mean (95% CI)**	**Mean (95% CI)**	**Mean (95% CI)**	**Mean (95% CI)**	**Mean (95% CI)**	
All	-0,8 (−1,2-(−0,3))	-0,6 (− 1,7-0,05))	-2,4 (−4,3-(− 0,5))	-0,1 (− 1,9-(− 0,1))	−1,3 (−2,5-(− 0,22)	0,481
**Gender**						
Women	−0,3 (− 0,9-0,3)	− 1,1 (− 2,5-0,4)	−2,7 (− 4,6-(− 0,8))	−2,0 (− 3,1-(− 0,9))	− 1,3 (− 2,7-0,08)	0,022
Men	−1,2 (− 2,0-(− 0,5))	−0,1 (− 1,7-1,5)	− 2,0 (−5,6 − 1,6)	0,04 (−1,4-1,4)	-1,4 (−3,1 − 0,3)	0,414
**Age groups**						
18–49 years	−0,8 (−1,5-(− 0,2))	-0,4 (− 2,0-1,2)	−2,8 (− 5,5-(− 0,05))	− 0,7 (− 2,0-0,6)	− 1,3 (− 3,0 − 0,3)	0,933
50–69 years	−0,6 (− 1,3-0,02)	-0,8 (−2,2-0,6)	− 1,8 (− 4,3-0,6)	−1,5 (− 2,6-(− 0,4))	−1,5 (− 2,9-(− 0,1))	0,075

The weighted average scores and their 95% confidence intervals (CI). Scores ranged from − 5 (negative change in all five key lifestyle factors) to 5 (positive change in all five key lifestyle factor). The five key lifestyle factors: vegetable consumption, leisure-time physical activity, sleep problems and nightmares, alcohol consumption, smoking

^1^Age and gender adjusted linear trend based on generalized linear models

## Discussion

Overall, the aim of the present study was to examine temporal changes in the key health-related lifestyle factors and the accumulation of positive or negative changes in the same individuals in the Finnish adult population during the COVID-19 epidemic. The results showed that lifestyle changes have been on average more commonly unfavorable than favorable for health. Every sixth reported at least two negative changes in the key lifestyle factors during the epidemic. Slightly slower proportion, about every seventh, reported at least two positive changes. The deteriorated epidemic situation in the late 2020 and, on the other hand, prolonged epidemic predisposed to negative lifestyle changes.

The accumulation of multiple negative lifestyle changes in the same individuals may have remarkable effects on public health if the changes due the epidemic remain long-term or even permanent. It is well documented that unhealthy lifestyle is a major risk factor for premature death, several chronic diseases as well as for mental health problems [25, 26, 32, 33]. In general, the larger the number of unhealthy lifestyle factors, the higher the risk of adverse health outcomes. Unhealthy lifestyle can also lead to weight gain, causing overweight and obesity, which are major causes of co-morbidities and other health problems [34]. Unhealthy lifestyle factors are risk factors for burden disease, but they are also risk factors for the COVID-19 hospital admission, accounting for up to half of severe cases [35].

According to our knowledge, this is the first study conducted in Finland focusing on temporal lifestyle changes at different stages of the epidemic. Our results indicate that the observed changes in lifestyle during COVID-19 epidemic did not occur linearly in one direction or other but tended to fluctuate over time by following the prevailing epidemic situation in Finland. The most negative lifestyle changes were reported in late 2020. At that time, the epidemic situation deteriorated in Finland, which may explain that. On the other hand, also prolonged epidemic situation tended to have negative effects on lifestyle. The proportions of those decreased leisure-time physical activity and active commuting to work increased as the epidemic drags on. Further, in the older age group, the proportion of those having multiple negative changes in the key lifestyle factors tended to increase during the epidemic.

Our data support the previous findings that both negative and positive changes in individual lifestyle factors were observed during the COVID-19 epidemic [11, 20, 36–39]. Many cross-sectional studies among different populations have found decrease in leisure-time physical activity [12–15, 17, 18, 36–38, 40–44] during the COVID-19 epidemic, but also increase has been reported (e.g.,20). The prevalence of decreased (range 36–47% in different annual quarters) and increased leisure-time physical activity (17–31%) during the COVID-19 epidemic in Finland, are quite comparable to that reported since the onset of COVID-19 in Australia (49%, vs. 20%) and during the lockdown in France (53% vs. 19%) [20, 22]. Findings from longitudinal studies indicate that lockdown had a negative effect on the leisure-time physical activity especially physical active people have reduced exercise more than physical inactive people during the COVID-19 epidemic [16, 40]. In addition to leisure-time physical activity, we observed a clear reduction in active commuting to work, which is probably due to increase in remote work. This may lead to increased sedentary time and decreased total physical activity. However, it could be that decreasing physical activity when commuting to work, is compensated by increasing leisure-time physical activity.

We observed that every third has increased unhealthy snacking during the epidemic. Compared to the previous studies, this proportion was higher than in the French NutriNet-Santé cohort [20], but somewhat lower than among Danish, Lithuanian, and UK populations [10, 11, 15]. A plausible explanation for increased snacking during COVID-19 epidemic could be an increase in time spent at home [9, 10] due to the restrictions and the marked increase in remote work. The time spent at home during social isolation has been found to be significantly correlated with snacking [9]. It has been suggested that negative changes in eating habits are due to anxiety or boredom [45]. The COVID-19 epidemic has caused stress [46], and stress is also known factor to influence eating habits [47]. The lockdown has been found to be associated with increased emotional eating [20]. There is also gender-specific response to stress: women were more likely to use food to deal with stress and men were more likely to smoking or use alcohol [47]. Our study support finding observing that increased snacking was more prevalent among women, which is also line with previous study [9]. Large numbers of studies have shown negative changes also in other eating habits during the COVID-19 epidemic [12–15, 20], like reduced consumption of fresh fruits and vegetables [20]. Deschasaux-Tanguy et al. [20] studied a sample of > 37,000 adults form the French web-based NutriNet-Santé cohort study where the aim was to characterize and cluster changes in lifestyle during the COVID-19 lockdown in France, showed decrease in consumption on fruits (18%) and vegetables (22%) during the COVID-19 epidemic, which were quite high proportions compared to our results (5–9%, 6–10%). In contrast, we observed some positive changes in the consumption of vegetables, fruits and berries. Also, Rodriquez-Perez et al. [48] observed the improvement of eating habits during the COVID-19 epidemic confinement in the Spanish population. However, the findings from cross-sectional studies on consumption of vegetables, fruits and berries are inconsistent [34, 49]. Due to the methodological differences, findings from the various studies cannot be directly compared. In some countries, the limited availability of fruits and vegetables and restricted food store opening during the epidemic [13] could have affected in the consumption of fruits and vegetables and due to that the results are not very comparable between different countries.

We observed that up to one fifth of respondents reported increased sleep problems due to the COVID-19. A meta-analysis involving a total of 54,231 respondents from 13 countries, showed that the prevalence of sleep problems was almost 36% among different populations during COVID-19 epidemic [23]. It has been suggested that some individuals have benefited from not having early morning commitments by reducing work or school schedules and their sleep quality improved, but others sleep quality deteriorated due to stress [50]. Our data shows that women were more likely to report increased sleep problems and nightmares than men, which could be due to gender specific response to stress and its effects on sleep. Further, results may reflect gender differences in sleep problems in the population overall. It is widely known that women suffer sleep problems more often than men [51]. In addition to stress, several other factors may have affected sleep. The combined effect of anxiety, stress and depression could be responsible for sleep problems during the Covid-19 epidemic [52, 53]**.** During the lockdown social isolation, family support, social support and social capital may also affect sleep [52, 54].

Previous cross-sectional studies have been showed increased alcohol consumption [9, 17–19] and smoking [17, 18] during the COVID-19 epidemic. The findings of the present study do not support those results showing decreased alcohol consumption and no changes in smoking during the study period. This may be due to differences between studied population and methodological or study settings differences. In particular, alcohol consumption may be under-reported.

In general, possible reasons for lifestyle changes have been generally suggested in several studies. Having more time available during the epidemic is the most cited reason for changing lifestyle [38]. In addition to that, the COVID-19 epidemic causes stress, which could modify the lifestyle changes during the epidemic [22]. It has been suggested that increased social isolation, loneliness, boredom, anxiety and depression generated by epidemic might have played major roles in lifestyle changes [55]. In the Swedish population, for example, unfavorable changes in lifestyle were associated with mental health problems [21]. On the other hand, it is also noteworthy that both our study and the previous studies (e.g., 10, 21) have shown that for several lifestyle factors the proportion of those who have not changed their habits at all during the epidemic is quite high. Thus, identification of those population groups that have been particularly vulnerable to unfavorable changes is essential to strengthen their resilience and to prevent the acceleration of health inequalities. The results of the present study emphasize the need to support healthy lifestyle at the population level and are useful to plan and target health promotion actions efficiently. Further, our observations that deteriorated epidemic situation and, on the other hand, prolonged epidemic predisposed to negative lifestyle changes are important for preparing for future epidemics. However, further investigation is needed to address long-term consequences of COVID-19 epidemic on lifestyle with accurate methodology and representative study samples. On the public health perspective, it is important to examine whether these changes are maintained over time and identify the factors that contribute to changes and their accumulation in the same individuals. The lifestyle factors studied in the present study are major risk factors for several chronic diseases as well as mental health problems [25, 33]. Thus, unfavorable development in them may increase the burden and costs of healthcare system and decrease quality of life and functional and working capacity at the individual level.

## Methodological considerations

The major strength of this study lies in the opportunity to use several time points to examine lifestyle changes over time. We were also able to study a wide variety of lifestyle factors enabling to study the accumulation of lifestyle changes during the epidemic. However, some limitations need to be acknowledged. First, we do not have pre-epidemic information on lifestyle of the respondents. The initial situation of lifestyle factors could affect interpretation of results. For example, physically active person can decrease physical activity and still move according to the recommendations. In that case, decreasing physical activity will not have the same adverse effects on health as if already physically inactive person decreases physical activity. Second, the non-response of the study was rather high (68%) and increasing over time, possibly weakening the representativeness and generalizability of results to the whole population and compromising the results of the trend tests. Unfavorable changes observed in alcohol consumption and smoking were minor which may be partly due that heavy drinkers and smokers are prone to non-response. However, the results were weighted according to registry-based information on age, gender, sampling date and geographical area to minimize the impact of non-response and attrition. Finally, in the present study, we only focused on health-related lifestyle factors. Many other factors, especially social factors such as feelings of loneliness and changes in social networks, may have impacted remarkably on health and wellbeing during the epidemic.

## Conclusions

This study provides novel evidence suggesting the impact of the COVID-19 pandemic on temporal changes in lifestyle habits are diverse being more often unfavorable than favorable for health. This study showed the accumulation of multiple negative changes in the key lifestyle factors in one-sixth of Finnish adults during the COVID-19 epidemic. The most common individual negative changes were an increase in snacking and sleep problems and decrease in leisure-time physical activity and active commuting to work. The deteriorated epidemic situation in the late 2020 and, on the other hand, prolonged epidemic predisposed to negative lifestyle changes. From a public health perspective, further studies are important to estimate whether these changes are maintained over time. It is also important to identify the factors that contribute to unfavorable changes, because unfavorable lifestyle factors predispose to weight gain and morbidity. Public health authorities should promote the adoption of healthy lifestyles to reduce long-term effects the COVID-19 epidemic has had on health and welfare.

## Supplementary Information

**Additional file 1: Supplementary Table 1.** Characteristics of the respondents and non-respondents (unweighted values).

**Additional file 2: Supplementary Table 2.** Changes in lifestyle by gender- and age-groups during the COVID-19 epidemic (from April 2020 to June 2021).

## Data Availability

Due to data protection reasons personal data cannot be publicly available. The data controller is Finnish Institute for Health and Welfare. Access to confidential data requires permission to handle the data, signed non-disclosure agreement as well as collaboration agreement with Finnish Institute for Health and Welfare. For more information, please contact: koronaserotutkimus@thl.fi.
