# Supplementary material for: Temporal changes in health-related lifestyle during the COVID-19 epidemic in Finland – a series of cross-sectional surveys
Source: BMC Public Health. 2022 Nov 19;22:2130. doi: 10.1186/s12889-022-14574-y (PMC9675975; doi:10.1186/s12889-022-14574-y)
Supplement: Supplementary file 1 — Additional file 1: Supplementary Table 1. Characteristics of the respondents and non-respondents (unweighted values). [file 12889_2022_14574_MOESM1_ESM.docx]

Supplementary table 1. Characteristics of the respondents and non-respondents (unweighted values).

|  | | | **Respondents,n** | **Non-respondents, n** | **Prevalence of respondents, %** | **Prevalence of non-respondents, %** | **Response rate, %** |
| --- | --- | --- | --- | --- | --- | --- | --- |
| **Total** | | | 5654 | 11938 |  |  | 32.1 |
| **Gender** | | |  |  |  |  |  |
|  | Men | | 2219 | 6541 | 39.2 | 54.8 | 25.3 |
|  | Women | | 3435 | 5397 | 60.8 | 45.2 | 38.9 |
| **Age group, years** | | |  |  |  |  |  |
|  | 18–29 | | 799 | 3288 | 14.1 | 27.5 | 19.5 |
|  | 30–39 | | 1100 | 2541 | 19.5 | 21.3 | 30.2 |
|  | 40–49 | | 1116 | 2177 | 19.7 | 18.2 | 33.9 |
|  | 50–59 | | 1387 | 2081 | 24.5 | 17.4 | 40 |
|  | 60–69 | | 1252 | 1851 | 22.1 | 15.5 | 40.3 |
| **Geographical area**^1^ | | |  |  |  |  |  |
|  | Helsinki | | 3013 | 6279 | 53.3 | 52.6 | 32.4 |
|  | Turku | | 625 | 1449 | 11.1 | 12.1 | 30.1 |
|  | Tampere | | 821 | 1513 | 14.5 | 12.7 | 35.2 |
|  | Kuopio | | 581 | 1201 | 10.3 | 10.1 | 32.6 |
|  | Oulu | | 614 | 1496 | 10.9 | 12.5 | 29.1 |
| **Education, years** | | |  |  |  |  |  |
|  | | 0–12 | 1703 | NA | 30.7 | NA | NA |
|  | | 13–16 | 1705 | NA | 30.7 | NA | NA |
|  | | 17+ | 2146 | NA | 38.6 | NA | NA |
| **Living alone** | | |  |  |  |  |  |
|  | | Yes | 996 | NA | 20.1 | NA | NA |
|  | | No | 3956 | NA | 79.9 | NA | NA |
| **Sampling date**^2^ | | |  |  |  |  |  |
|  | | 2020-04-01 | 3082 | 4035 | 54.5 | 33.8 | 43.3 |
|  | | 2020-07-01 | 639 | 1709 | 11.3 | 14.3 | 27.2 |
|  | | 2020-10-01 | 702 | 2476 | 12.4 | 20.7 | 22.1 |
|  | | 2021-01-01 | 749 | 2006 | 13.2 | 16.8 | 27.2 |
|  | | 2021-04-01 | 482 | 1712 | 8.5 | 14.3 | 22 |

^1^ University hospital; ^2^ Samples during 3 month periods (starting dates of the periods)
