# Supplementary material for: Temporal changes in health-related lifestyle during the COVID-19 epidemic in Finland – a series of cross-sectional surveys
Source: BMC Public Health. 2022 Nov 19;22:2130. doi: 10.1186/s12889-022-14574-y (PMC9675975; doi:10.1186/s12889-022-14574-y)
Supplement: Supplementary file 2 — Additional file 2: Supplementary Table 2. Changes in lifestyle by gender- and age-groups during the COVID-19 epidemic (from April 2020 to June 2021). [file 12889_2022_14574_MOESM2_ESM.docx]

**Supplementary table 2.** Changes in lifestyle by gender- and age-groups during the COVID-19 epidemic (from April 2020 to June 2021)**.**

| **Lifestyle factors**  **(n=5655)** | **April-June**  **2020** | **July-September 2020** | **October-December**  **2020** | | **January-March 2021** | | **April-June**  **2021** | | **p-trend^1^** | |  |
| --- | --- | --- | --- | --- | --- | --- | --- | --- | --- | --- | --- |
|  | % (95% CI) | % (95% CI) | % (95% CI) | | % (95% CI) | | % (95% CI) | |  | |  |
| **Consumption of vegetables** | | | |  | |  | |  | |  | |
| **Decreased (n=425)** | | | | | | | | | | |  |
| **Gender** |  |  |  | |  | |  | |  | |  |
| Men | 10,6 (8,4-12,8) | 7,1 (2,6-11,6) | 10,1 (0,9-19,3) | | 7,3 (0,4-10,6) | | 5,9 (1,8-10,0) | | 0,007 | |  |
| Women | 8,4 (6,8-10,0) | 3,7 (1,0-6,4) | 5,6 (0,7-10,5) | | 7,5 (5,0-10,0) | | 8,6 (4,9-12,3) | | 0,266 | |  |
| **Age groups** |  |  |  | |  | |  | |  | |  |
| 18-49 years | 10,6 (8,8-12,4) | 6,0 (2,3-9,7) | 7,6 (0,9-14,3) | | 7,7 (5,0-10,4) | | 6,2 (2,7-9,7) | | 0,002 | |  |
| 50-69 years | 7,1 (5,3-8,9) | 4,6 (0,9-8,3) | 8,2 (0,4-16,0) | | 6,8 (3,7-9,9) | | 9,7 (4,8-14,6) | | 0,857 | |  |
| **Increased (n=901)** | | | | | | | | | | |  |
| **Gender** |  |  |  | |  | |  | |  | |  |
| Men | 11,2 (9,0-13,4) | 16,1 (9,6-22,6) | 15,8 (6,0-25,6) | | 20,6 (15,3-25,9) | | 10,4 (5,1-15,7) | | 0,188 | |  |
| Women | 16,9 (14,5-19,3) | 18,2 (12,5-23,9) | 9,6 (3,9-15,3) | | 18,3 (14,4-22,2) | | 18,9 (13,6-24,2) | | 0,041 | |  |
| **Age groups** |  |  |  | |  | |  | |  | |  |
| 18-49 years | 13 (11,0-15,0) | 15,1 (9,4-20,8) | 11,1 (4,2-18,0) | | 19,0 (14,9-23,1) | | 13,3 (8,6-18,0) | | 0,09 | |  |
| 50-69 years | 16,3 (13,6-19,0) | 20,7 (14,0-27,4) | 15,6 (6,0-25,2) | | 20,5 (15,4-25,6) | | 16,9 (10,6-23,2) | | 0,088 | |  |
|  |  |  |  | |  | |  | |  | |  |
| **Consumption of fruit and berries** | | | | | | | | | | | |
| **Decreased (n=382)** | | | | | | | | | | |  |
| **Gender** |  |  |  | |  | |  | |  | |  |
| Men | 7,5 (5,5-9,5) | 3,0 (5,7-10,1) | 10,1 (0,9-19,3) | | 9,0 (5,1-12,9) | | 5,8 (2,1-9,5) | | 0,438 | |  |
| Women | 7,3 (5,7-8,9) | 6,4 (2,9-9,9) | 5,6 (0,7-10,5) | | 6,6 (4,1-9,1) | | 12,3 (7,8-16,8) | | 0,568 | |  |
| **Age groups** |  |  |  | |  | |  | |  | |  |
| 18-49 years | 8,2 (6,6-9,8) | 4,2 (1,5-6,9) | 6,9 (0,4-13,4) | | 9,2 (6,1-12,3) | | 7,9 (4,4-11,4) | | 0,564 | |  |
| 50-69 years | 5,6 (3,8-7,4) | 5,2 (1,7-8,7) | 9,5 (1,5-17,5) | | 4,5 (1,8-7,2) | | 10,9 (5,8-16,0) | | 0,577 | |  |
| **Increased (n=1020)** | | | | | | | | | | |  |
| **Gender** |  |  |  | |  | |  | |  | |  |
| Men | 14,0 (11,6-16,4) | 18,0 (11,5-24,5) | 12,4 (3,6-21,2) | | 16,8 (12,3-21,3) | | 9,2 (4,1-14,3) | | 0,972 | |  |
| Women | 17,2 (15,4-20,2) | 22,6 (16,5-28,7) | 8,3 (3,0-13,6) | | 22,1 (18,0-26,2) | | 22,2 (16,7-27,7) | | 0,074 | |  |
| **Age groups** |  |  |  | |  | |  | |  | |  |
| 18-49 years | 15,5 (13,3-17,7) | 17,3 (11,4-23,2) | 9,2 (2,9-15,5) | | 17,4 (13,7-21,1) | | 13,1 (8,6-17,6) | | 0,737 | |  |
| 50-69 years | 16,7 (14,0-19,4) | 25,2 (18,1-32,3) | 12,4 (4,2-20,6) | | 24,2 (18,9-29,5) | | 20,7 (14,0-27,4) | | 0,01 | |  |
|  |  |  |  | |  | |  | |  | |  |
| **Snacking** |  |  |  | |  | |  | |  | |  |
| **Decreased (n=291)** | | | | | | | | | | |  |
| **Gender** |  |  |  | |  | |  | |  | |  |
| Men | 4,2 (2,8-5,6) | 1,8 (-0,4-4,0) | 8,6 (1,3-15,9) | | 8,4 (4,7-12,1) | | 5,0 (1,1-8,9) | | 0,026 | |  |
| Women | 4,4 (3,2-5,6) | 7,8 (3,5-12,1) | 1,9 (-0,6-4,4) | | 5,3 (3,1-7,5) | | 5,6 (2,5-8,7) | | 0,686 | |  |
| **Age groups** |  |  |  | |  | |  | |  | |  |
| 18-49 years | 3,8 (2,6-5,0) | 5,7 (2,4-9,0) | 4,7 (0,2-9,2) | | 6,4 (3,7-9,1) | | 5,1 (2,0-8,2) | | 0,047 | |  |
| 50-69 years | 5,3 (3,5-7,1) | 2,9 (0,2-5,6) | 5,9 (-0,8-12,6) | | 7,8 (4,5-11,1) | | 5,8 (1,9-9,7) | | 0,539 | |  |
| **Increased (n=1680)** | | | | | | | | | | |  |
| **Gender** |  |  |  | |  | |  | |  | |  |
| Men | 28,8 (25,5-32,1) | 27,4 (19,4-35,4) | 35,8 (21,7-49,9) | | 27,5 (21,6-33,4) | | 25,2 (17,6-32,8) | | 0,169 | |  |
| Women | 36,8 (33,9-39,7) | 34,1 (27,0-41,2) | 39,8 (29,4-50,2) | | 40,6 (35,7-45,5) | | 42,3 (35,6-49,0) | | 0,928 | |  |
| **Age groups** |  |  |  | |  | |  | |  | |  |
| 18-49 years | 37,3 (34,4-40,2) | 35,6 (28,2-43,0) | 43,6 (32,2-55,0) | | 38,6 (33,5-43,7) | | 35,0 (28,3-41,7) | | 0,195 | |  |
| 50-69 years | 22,7 (19,8-25,6) | 21,6 (14,9-28,3) | 26,0 (14,6-37,4) | | 23,3 (18,2-28,4) | | 27,7 (20,4-35,0) | | 0,825 | |  |
|  |  |  |  | |  | |  | |  | |  |
| **Consumption of alcohol** | | | | | | | | | | | |
| **Decreased (n=726)** | | | | | | | | | | |  |
| **Gender** |  |  |  | |  | |  | |  | |  |
| Men | 19,6 (16,5-22,7) | 9,7 (4,4-15,0) | 15,9 (5,4-5,3) | | 23,8 (17,7-29,9) | | 22,4 (14,8-30,0) | | 0,025 | |  |
| Women | 13,8 (11,6-16,0) | 8,4 (3,9-12,9) | 10,9 (3,8-18,0) | | 20,4 (16,1-24,7) | | 15,2 (10,3-20,1) | | 0,155 | |  |
| **Age groups** |  |  |  | |  | |  | |  | |  |
| 18-49 years | 20,5 (18,0-23,0) | 11,8 (6,7-16,9) | 15,3 (6,9-23,7) | | 27,3 (22,4-32,2) | | 21,5 (15,4-27,6) | | 0,009 | |  |
| 50-69 years | 8,6 (6,4-10,8) | 4,3 (1,0-7,6) | 9,1 (1,3-16,9) | | 9,9 (6,2-13,6) | | 12,6 (7,1-18,1) | | 0,694 | |  |
| **Increased (n=392)** | | | | | | | | | | |  |
| **Gender** |  |  |  | |  | |  | |  | |  |
| Men | 8,9 (6,7-11,1) | 8,4 (3,9-12,9) | 9,4 (0,6-18,2) | | 6,2 (3,3-9,1) | | 9,7 (4,6-14,8) | | 0,46 | |  |
| Women | 7,2 (5,6-8,8) | 10,6 (6,1-15,1) | 1,1 (-1,1-3,3) | | 7,3 (4,8-9,8) | | 6,6 (3,1-10,1) | | 0,317 | |  |
| **Age groups** |  |  |  | |  | |  | |  | |  |
| 18-49 years | 9,6 (7,8-11,4) | 8,0 (3,9-12,1) | 3,6 (-1,9-9,1) | | 7,6 (5,1-10,1) | | 9,4 (5,3-13,5) | | 0,222 | |  |
| 50-69 years | 4,7 (3,1-6,3) | 12,0 (6,7-17,3) | 8,3 (0,5-16,1) | | 4,9 (2,2-7,6) | | 5,3 (1,6-9,0) | | 0,84 | |  |
|  |  |  |  | |  | |  | |  | |  |
| **Smoking** |  |  |  | |  | |  | |  | |  |
| **Decreased (n=201)** | | | | | | | | | | |  |
| **Gender** |  |  |  | |  | |  | |  | |  |
| Men | 4,9 (3,3-6,5) | 0,6 (-0,6-1,8) | 3,2 (-1,3-7,7) | | 6,7 (3,4-10,0) | | 4,2 (0,7-7,7) | | 0,858 | |  |
| Women | 3,2 (2,2-4,2) | 3,7 (0,6-6,8) | 1,1 (-1,1-3,3) | | 4,6 (2,4-6,8) | | 1,4 (0,0-2,8) | | 0,817 | |  |
| **Age groups** |  |  |  | |  | |  | |  | |  |
| 18-49 years | 4,0 (2,8-5,2) | 2,2 (0-4,4) | 2,1 (-0,8-5,0) | | 6,5 (3,8-9,2) | | 2,4 (-0,1-4,9) | | 0,688 | |  |
| 50-69 years | 4,1 (2,5-5,7) | 2,0 (-0,4-4,4) | 2,2 (-2,1-6,5) | | 3,5 (1,1-5,9) | | 4,4 (0,9-7,9) | | 0,408 | |  |
| **Increased (n=136)** | | | | | | | | | | |  |
| **Gender** |  |  |  | |  | |  | |  | |  |
| Men | 2,2 (1,0-3,4) | 1,2 (-0,4-2,8) | 5,4 (-2,2-13,0) | | 1,5 (-0,1-3,1) | | 3 (-0,1-6,1) | | 0,293 | |  |
| Women | 3,1 (1,9-4,3) | 4,3 (1,4-7,2) | 1,1 (-0,1-3,3) | | 2,5 (0,9-4,1) | | 1,2 (-0,2-2,6) | | 0,024 | |  |
| **Age groups** |  |  |  | |  | |  | |  | |  |
| 18-49 years | 3,1 (2,1-4,1) | 3,1 (0,7-5,5) | 4,7 (-1,0-10,4) | | 2,5 (0,9-4,1) | | 2,5 (0,1-4,9) | | 0,87 | |  |
| 50-69 years | 1,5 (0,7-2,3) | 1,9 (-0,3-4,1) | 0 (0-0) | | 1,0 (-0,2-2,2) | | 1,3 (-0,5-3,1) | | 0,058 | |  |
|  |  |  |  | |  | |  | |  | |  |
| **Sleep problems and nightmares** | | | | | | | | | | | |
| **Decreased (n=84)** |  |  |  | |  | |  | |  | |  |
| **Gender** |  |  |  | |  | |  | |  | |  |
| Men | 0,7 (0,1-1,3) | 2,4 (0,0-4,8) | 3,6 (-1,3-8,5) | | 1,1 (-0,1-2,3) | | 1,9 (0,1-3,7) | | 0,302 | |  |
| Women | 20,0 (1,2-2,8) | 0,9 (-0,3-2,1) | 2,9 (-1,2-7,0) | | 1,5 (0,3-2,7) | | 2,3 (0,1-4,5) | | 0,569 | |  |
| **Age groups** |  |  |  | |  | |  | |  | |  |
| 18-49 years | 1,2 (0,6-1,8) | 1,0 (-0,6-2,6) | 3,7 (-0,6-8,0) | | 1,3 (0,3-2,3) | | 2,2 (0,4-4,0) | | 0,562 | |  |
| 50-69 years | 1,7 (0,7-2,7) | 2,9 (0,2-5,6) | 2,1 (-0,2-6,2) | | 1,4 (0-2,8) | | 1,7 (-0,7-4,1) | | 0,684 | |  |
| **Increased (n=959)** | | | | | | | | | | |  |
| **Gender** |  |  |  | |  | |  | |  | |  |
| Men | 12,4 (10,0-14,8) | 9,7 (4,2-15,2) | 21,2 (9,8-32,6) | | 11,1 (7,2-15,0) | | 10,0 (5,1-14,9) | | 0,479 | |  |
| Women | 20,1 (17,7-22,5) | 16,9 (11,2-22,6) | 22,8 (14,2-31,4) | | 23,4 (19,1-27,7) | | 21,2 (15,9-26,5) | | 0,722 | |  |
| **Age groups** |  |  |  | |  | |  | |  | |  |
| 18-49 years | 17,2 (15,0-19,4) | 14,4 (8,9-19,9) | 18,2 (9,6-26,8) | | 18,5 (14,6-22,4) | | 13,6 (9,1-18,1) | | 0,335 | |  |
| 50-69 years | 13,8 (11,3-16,3) | 10,6 (5,7-15,5) | 30,1 (17,9-42,3) | | 14,3 (10,0-18,6) | | 19,0 (12,7-25,3) | | 0,386 | |  |
|  |  |  |  | |  | |  | |  | |  |
| **Leisure-time physical activity** | | | | | | | | | | | |
| **Decreased (n=2046)** | | | | | | | | | | |  |
| **Gender** |  |  |  | |  | |  | |  | |  |
| Men | 38,5 (35,0-42,0) | 34,9 (26,3-43,5) | 43,3 (29,2-57,4) | | 46,1 (39,6-52,6) | | 44,8 (36,4-53,2) | | 0,142 | |  |
| Women | 32,4 (29,7-35,1) | 33,7 (26,8-40,6) | 45,2 (34,6-55,8) | | 48,1 (43,0-53,2) | | 45,2 (38,5-51,9) | | <0,001 | |  |
| **Age groups** |  |  |  | |  | |  | |  | |  |
| 18-49 years | 37,1 (34,2-40,0) | 37,7 (30,1-45,3) | 47,3 (36,1-58,5) | | 48,1 (42,8-53,4) | | 45,3 (38,4-52,2) | | <0,001 | |  |
| 50-69 years | 32 (28,7-35,3) | 28,5 (21,2-35,8) | 38,1 (25,4-50,8) | | 45,0 (38,7-51,3) | | 44,1 (35,9-52,3) | | 0,001 | |  |
| **Increased (n=1462)** | | | | | | | | | | |  |
| **Gender** |  |  |  | |  | |  | |  | |  |
| Men | 26,4 (23,3-29,5) | 21,4 (14,7-28,1) | 24,7 (13,3-36,1) | | 20,3 (15,0-25,6) | | 22,1 (14,8-29,4) | | 0,172 | |  |
| Women | 35,2 (32,3-38,1) | 29,3 (22,6-36,0) | 10,7 (4,2-17,2) | | 24,1 (19,8-28,4) | | 28,8 (22,5-35,1) | | <0,001 | |  |
| **Age groups** |  |  |  | |  | |  | |  | |  |
| 18-49 years | 32,9 (30,2-35,6) | 24,3 (18,0-30,6) | 14,9 (7,1-22,7) | | 23,6 (19,3-27,9) | | 25,2 (19,1-31,3) | | 0,001 | |  |
| 50-69 years | 26,1 (23,0-29,2) | 26,6 (19,3-33,9) | 22,6 (11,2-34,0) | | 18,9 (14,0-23,8) | | 24,9 (17,5-32,3) | | 0,071 | |  |
|  |  |  |  | |  | |  | |  | |  |
| **Active commuting to work** | | | | | | | | | | | |
| **Decreased (n=1553)** | | | | | | | | | | |  |
| **Gender** |  |  |  | |  | |  | |  | |  |
| Men | 40,2 (36,7-43,7) | 29,1 (21,1-37,1) | 34,7 (21,2-48,2) | | 41,0 (34,5-47,5) | | 37,6 (29,4-45,8) | | 0,29 | |  |
| Women | 42,4 (39,5-45,2) | 26,5 (20,0-33,0) | 35,6 (25,4-45,8) | | 44,1 (39,0-49,2) | | 38,1 (31,4-44,8) | | 0,01 | |  |
| **Age groups** |  |  |  | |  | |  | |  | |  |
| 18-49 years | 44,2 (41,3-47,1) | 30,1 (23,0-37,2) | 35,8 (25,0-46,6) | | 44,3 (39,2-49,4) | | 39,1 (32,2-46,0) | | 0,03 | |  |
| 50-69 years | 34,8 (31,3-38,3) | 24,0 (17,1-30,9) | 33,8 (21,5-46,1) | | 38,7 (32,6-44,8) | | 34,6 (26,6-42,6) | | 0,23 | |  |
| **Increased (n=275)** | | | | | | | | | | |  |
| **Gender** |  |  |  | |  | |  | |  | |  |
| Men | 2,6 (1,4-3,8) | 8,1 (3,0-13,2) | 10 (2,2-17,8) | | 5,2 (2,3-8,1) | | 3,2 (0,3-6,1) | | 0,354 | |  |
| Women | 8,5 (6,7-10,3) | 8,6 (4,5-12,7) | 12,1 (5,0-19,2) | | 6,9 (4,4-9,4) | | 10,5 (6,4-14,6) | | 0,967 | |  |
| **Age groups** |  |  |  | |  | |  | |  | |  |
| 18-49 years | 5,6 (4,2-7,0) | 10,0 (5,1-14,9) | 11,6 (4,7-18,5) | | 6,0 (3,6-8,4) | | 6,8 (3,7-9,9) | | 0,813 | |  |
| 50-69 years | 5,3 (3,7-6,9) | 5,4 (1,9-8,9) | 10,0 (2,4-17,6) | | 6,1 (3,2-9,0) | | 6,0 (2,1-9,9) | | 0,512 | |  |

Weighted prevalences (%) and 95 % confidence intervals (CI).

**^1^**  Age and gender adjusted linear trend based on generalized linear models.
